# Assessing Osteolytic Lesion Size on Sequential CT Scans Is a Reliable Study Endpoint for Bone Remineralization in Newly Diagnosed Multiple Myeloma

**DOI:** 10.3390/cancers15154008

**Published:** 2023-08-07

**Authors:** Jan-Peter Grunz, Andreas Steven Kunz, Freerk T. Baumann, Dirk Hasenclever, Malte Maria Sieren, Stefan Heldmann, Thorsten Alexander Bley, Hermann Einsele, Stefan Knop, Franziska Jundt

**Affiliations:** 1 Department of Diagnostic and Interventional Radiology, University Hospital Würzburg, Oberdürrbacher Straße 6, 97080 Würzburg, Germany; kunz_a@ukw.de (A.S.K.); bley_t@ukw.de (T.A.B.); 2 Department I of Internal Medicine, Center for Integrated Oncology Aachen Bonn Cologne Dusseldorf, University Hospital of Cologne, Kerpener Straße 62, 50937 Cologne, Germany; freerk.baumann@uk-koeln.de; 3 Institute for Medical Informatics, Statistics and Epidemiology, University of Leipzig, Härtelstraße 16–18, 04107 Leipzig, Germany; dirk.hasenclever@imise.uni-leipzig.de; 4 Department of Radiology and Nuclear Medicine, University Hospital Schleswig-Holstein, Ratzeburger Allee 160, 23562 Lübeck, Germany; malte.sieren@uksh.de; 5 Institute of Interventional Radiology, University Hospital Schleswig-Holstein, Ratzeburger Allee 160, 23562 Lübeck, Germany; 6 Fraunhofer Institute for Digital Medicine MEVIS, Maria-Goeppert-Straße 3, 23562 Lübeck, Germany; stefan.heldmann@mevis.fraunhofer.de; 7 Department of Internal Medicine II, University Hospital Würzburg, Oberdürrbacher Straße 6, 97080 Würzburg, Germany; einsele_h@ukw.de (H.E.); stefan.knop@klinikum-nuernberg.de (S.K.); jundt_f@ukw.de (F.J.); 8 Department of Internal Medicine, Klinikum Nürnberg Nord, Prof.-Ernst-Nathan-Str. 1, 90419 Nürnberg, Germany

**Keywords:** multiple myeloma, bone remineralization, computed tomography, whole-body imaging

## Abstract

**Simple Summary:**

While MRI is primarily used for vitality analysis in multiple myeloma, the detection of osteolytic manifestations in the mineralized bone is performed on CT scans. For this study based on a homogenous sample of 20 patients with newly diagnosed multiple myeloma, we hypothesized that sequential CT studies can be used to validate remineralization quantitatively and qualitatively as a measure of treatment response. After six cycles of standardized induction therapy with the anti-SLAMF7 antibody elotuzumab in combination with carfilzomib, lenalidomide, and dexamethasone (E-KRd), we were able to record a substantial lesion size decrease associated with the formation of trabecular sclerosis in the majority of responding manifestations.

**Abstract:**

Multiple myeloma (MM) frequently induces persisting osteolytic manifestations despite hematologic treatment response. This study aimed to establish a biometrically valid study endpoint for bone remineralization through quantitative and qualitative analyses in sequential CT scans. Twenty patients (seven women, 58 ± 8 years) with newly diagnosed MM received standardized induction therapy comprising the anti-SLAMF7 antibody elotuzumab, carfilzomib, lenalidomide, and dexamethasone (E-KRd). All patients underwent whole-body low-dose CT scans before and after six cycles of E-KRd. Two radiologists independently recorded osteolytic lesion sizes, as well as the presence of cortical destruction, pathologic fractures, rim and trabecular sclerosis. Bland–Altman analyses and Krippendorff’s α were employed to assess inter-reader reliability, which was high for lesion size measurement (standard error 1.2 mm) and all qualitative criteria assessed (α ≥ 0.74). After six cycles of E-KRd induction, osteolytic lesion size decreased by 22% (*p* < 0.001). While lesion size response did not correlate with the initial lesion size at baseline imaging (Pearson’s r = 0.144), logistic regression analysis revealed that the majority of responding osteolyses exhibited trabecular sclerosis (*p* < 0.001). The sum of osteolytic lesion sizes on sequential CT scans defines a reliable study endpoint to characterize bone remineralization. Patient level response is strongly associated with the presence of trabecular sclerosis.

## 1. Introduction

Multiple myeloma (MM) is a malignant disease of the bone marrow in which the excessive proliferation of plasma cells occurs. MM constitutes the second most common hematologic neoplasm in the USA and Europe [1] and accounts for approximately 1% of all new cancer cases worldwide [2]. MM manifestations differ in the degree and pattern of bone marrow and extramedullary involvement [1,3]. The role of radiology in the assessment of patients with MM includes the detection of vital manifestations and their evaluation under treatment [4]. While whole-body MRI with diffusion-weighted imaging sequences is considered the reference standard for the diagnosis of diffuse and focal plasma cell infiltration in the bone marrow and the assessment of lesion vitality [5,6,7], the detection of osteolytic lesions in the mineralized bone is generally performed based on unenhanced low-dose whole-body CT [8]. Skeletal manifestations of MM can range from osteopenia to singular or diffuse osteolytic lesions (80–90% of patients) as a result of osteoblast inhibition and increased osteoclast activity [9].

Bisphosphonates inhibiting osteoclast activity and denosumab, a monoclonal antibody against the receptor activator of nuclear factor-kappa B ligand (RANKL), have been a pillar of bone-targeted therapy for many years [10,11]. However, the simultaneous decrease in osteoblast activity in MM commonly results in the persistence of osteolytic lesions even in patients with hematologic responses after treatment [9]. Recent therapy regimens employing proteasome inhibitors (e.g., carfilzomib) and immunomodulatory drugs (e.g., lenalidomide) have improved the prognosis of MM patients considerably [12]. Proteasome inhibitors in particular have been described to positively influence bone turnover due to osteoblastogenesis with the potential to induce the limited remineralization of osteolytic lesions [13,14,15]. More recently, the ELOQUENT-2 phase III trial indicated prolonged progression-free survival and a higher frequency of remission in MM patients that received the monoclonal antibody elotuzumab directed against the “signaling lymphocytic activation molecule family member 7” (SLAMF7) in addition to standard induction treatment [16]. In another recent phase II study, a therapy regimen combining elotuzumab with weekly carfilzomib, lenalidomide, and dexamethasone (E-KRd) was associated with durable hematologic treatment responses [17]. Despite promising results for overall disease outcome, the frequency and extent of bone remineralization after MM induction therapy has not been thoroughly investigated thus far. Bearing a substantial risk for pathologic fractures and the occurrence of adverse events, such as spinal cord compression or vertebral column instability, persistent focal osteolytic lesions must not be underestimated [9].

To define a reliable radiologic study endpoint, this investigation aimed to assess bone remineralization quantitatively and qualitatively in sequential whole-body low-dose CT scans of patients treated with six cycles of E-KRd as an induction therapy.

## 2. Materials and Methods

For this retrospective study, permission was obtained from the local institutional review board. The need for additional written informed consent was waived.

### 2.1. Study Population

Eligibility criteria for study inclusion comprised the following: age between 18 and 70 years, newly diagnosed multiple myeloma according to the International Myeloma Working Group (IMWG) updated criteria [18], induction therapy with six cycles of E-KRd within the DSMM XVII interventional phase III trial (ClinicalTrials.gov identifier: NCT03948035; therapy regimen summarized in Supplemental Table S1), as well as whole-body low-dose CT scans before and after six cycles of E-KRd treatment between September 2018 and February 2021. If not indicated otherwise, patients received either bisphosphonates (4 mg zoledronic acid every 4 weeks) or denosumab (120 mg every 4 weeks) as standard of care. For each patient, therapy response was assessed by standard serological and urine laboratory tests, as well as bone marrow punctures before and after treatment. Response was determined based on the current version of the IMWG consensus criteria [19].

### 2.2. Imaging Parameters

Whole-body low-dose CT scans were performed in craniocaudal orientation from the vertex to the proximal tibia metaphysis using a third-generation dual-source CT scanner (Somatom Force, Siemens Healthineers, Erlangen, Germany). Since the color-coded mapping of bone marrow infiltration was not in the scope of this study, both dual-energy (patient weight <90 kg: 90/Sn 150 kVp, patient weight ≥90 kg: 100/Sn 150 kVp) and single-energy examinations (100 or 120 kVp) were included in the analysis. Automatic tube current modulation (Care Dose 4D; Siemens Healthineers) was employed for all CT studies and no intravenous contrast medium was administered. The mean dose-length product and volume CT dose index of examinations were 928.5 ± 616.1 mGy × cm and 6.6 ± 4.4 mGy, respectively. Raw data were reconstructed with 3 mm slice thickness, 3 mm increment, and 512 × 512 matrix using standard-resolution bone, lung and soft tissue kernels.

### 2.3. Radiological Assessment of Treatment Response

Whole-body low-dose CT studies before and after E-KRd induction therapy were read in pairs by two independent radiologists with six and nine years of experience in oncologic imaging. Readers were tasked with identifying a maximum of ten representative osteolytic lesions per patient with short-axis diameters ≥5 mm on axial CT slices and quantifying the expanse of each lesion (short-axis x long-axis diameter (mm^2^)). The presence of cortical destruction, pathologic fractures, and rim and trabecular sclerosis was assessed dichotomously (present/absent). “Rim sclerosis” was defined as osteosclerotic alterations limited to the edges of a lesion, whereas “trabecular sclerosis” was recorded if osteosclerosis occurred within a lesion. A “pathologic fracture” was presumed if a fracture line or vertebral height loss was associated with an osteolytic lesion. For visualization purposes, color-coded subtraction maps were prepared with a variational image registration algorithm that combines rigid pre-alignment with non-linear deformable registration (PACS Viewer Application, Fraunhofer Institute for Digital Medicine MEVIS) [20]. All examinations were reviewed on standard radiological workstations with commercially available PACS software (Merlin, Phönix-PACS, Freiburg im Breisgau, Germany).

### 2.4. Statistics

**Figure 1:**
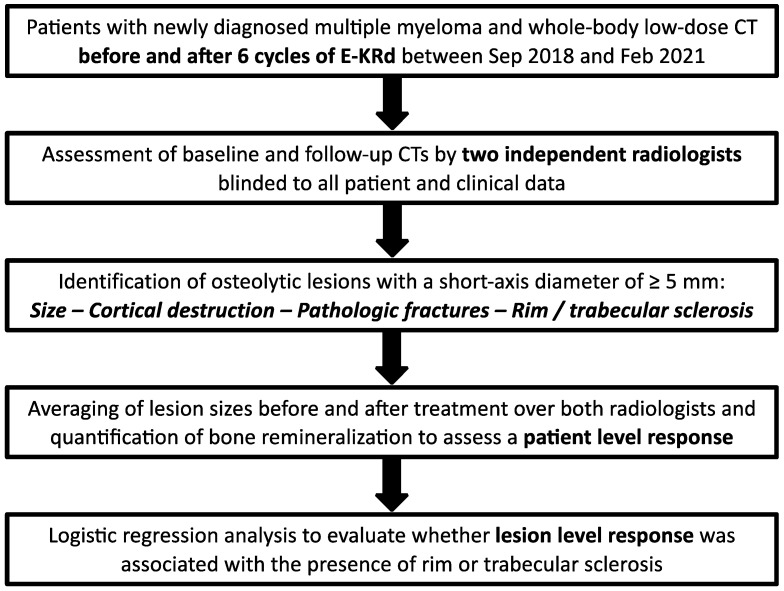
Flowchart illustrating the study design.

Since two radiologists independently assessed five different characteristics for each detected lesion (two-dimensional lesion size measurements and four qualitative features), a patient-level endpoint aggregated over all lesions was required. Being a metric variable, lesion size (reported as mean ± standard deviation) is easily summed up, which is why we decided to employ the four binary variables for validation, expecting that bone remineralization would reduce lesion size particularly in MM manifestations exhibiting rim or trabecular sclerosis. To identify an adequate error model for inter-reader differences in lesion size, a Bland–Altman analysis was performed on the linear, logarithmic, and square root scales. The reader concordance of binary features was described by crosstabulation, and Krippendorff’s α was computed with a 95% confidence interval to analyze inter-reader agreement for dichotomous criteria of response. Lesion sizes were then averaged over the two radiologists and bone remineralization response to treatment per lesion was quantified as a ratio. For a patient-level endpoint, we used the ratio of the sum of lesion sizes after and before treatment. Finally, logistic regression analysis was performed to evaluate whether lesion-level response was associated with the presence of rim or trabecular sclerosis. A flowchart illustrating the study design is presented in Figure 1.

## 3. Results

### 3.1. Patient Characteristics

The study sample consisted of 20 patients (seven women) with a mean age of 58 ± 8 years. The MM subtypes IgG kappa, IgG lambda, IgA kappa, and IgA lambda were recorded in 11, 5, 3, and 1 individuals, respectively. Employing the Revised International Staging System (R-ISS) [21], 11, 8, and 1 patients were categorized as R-ISS I, II, and III. The median bone marrow infiltration in the study population before commencement of E-KRd induction therapy was 50% (IQR 20–71%). The proportion of plasma cells decreased in all 20 patients after six cycles of treatment (median decrease 48%, IQR 19–66%). At the end of E-KRd induction therapy, the hematologic result was determined as “very good partial response” (VGPR) in 16 patients, “complete response” (CR) in 3 patients, and “stringent complete response” (sCR) in 1 patient. The mean time interval between whole-body low-dose CT scans was 221 ± 26 days. Individual patient characteristics are presented in Table 1.

### 3.2. Quantitative Lesion Characterization before and after Induction Therapy

**Figure 2:**
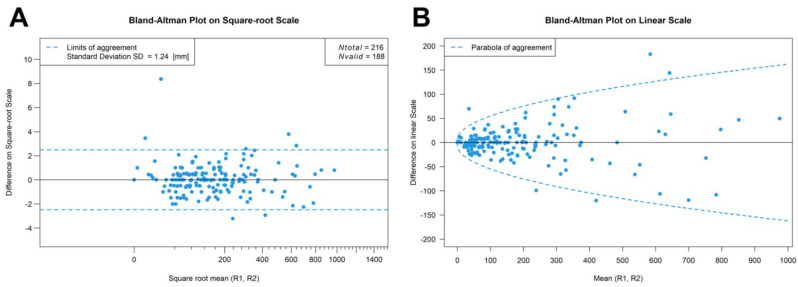
On the square root scale (**A**), inter-reader ratios are approximately independent of the overall lesion size. Using a square root scale corresponds to a model of constant additive errors in length measurements (mm). Combining baseline and follow-up imaging results, 188 total lesions were assessed by both radiologists. The dependence of the measurement error of a single lesion on the lesion size is illustrated on the linear scale (**B**).

**Figure 3:**
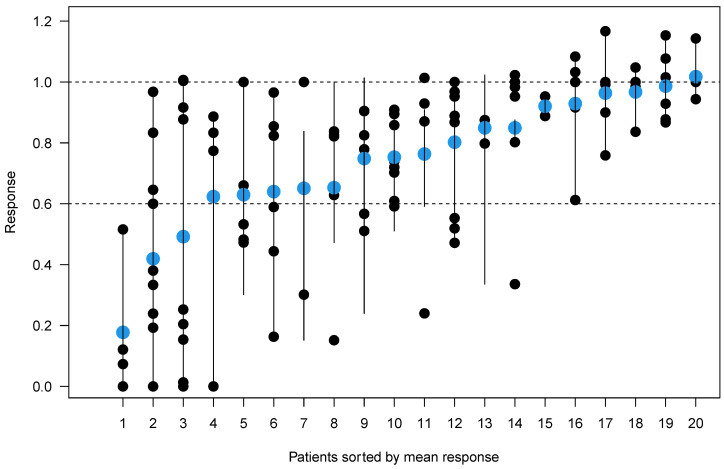
To offset the individual reader effects, we calculated averages from each pair of independent measurements. The stalactite plot shows patients on the *x*-axis sorted by their mean therapy response with all lesions in each patient plotted. The range of lesion responses is indicated as a connecting line and the response averages are represented as blue dots.

**Figure 4:**
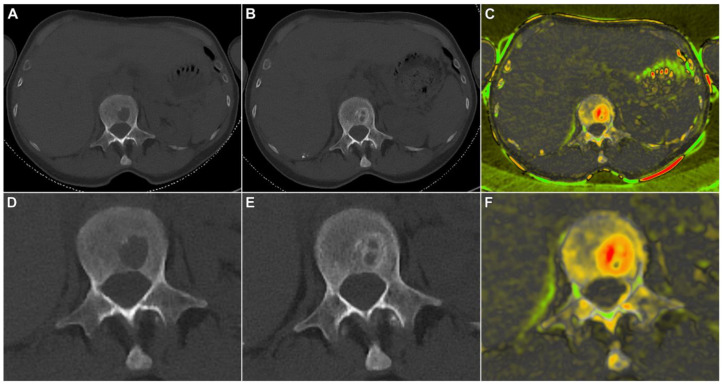
Depiction of therapy response in a 62-year-old woman with multiple myeloma (IgG kappa, R-ISS I). Baseline CT exhibited an osteolytic lesion in lumbar vertebra 1 (**A**,**D**) with trabecular sclerosis after six cycles of E-KRd (**B**,**E**). A color-coded subtraction map highlights the extent of remineralization within different parts of the lesion (**C**,**F**). Upper row: standard view. Lower row: zoomed image of the same slice.

A total of 108 different osteolytic lesions were recorded with readers 1 and 2 describing 103 and 99 lesions, respectively. Ninety-four lesions were assessed by both readers at baseline and follow-up imaging and used for further statistical evaluation. Measurement differences were independent of lesion size on the square root scale, indicating that discrepancies are due to one-dimensional measurement errors with a typical standard deviation of 1.2 mm. In contrast, measurement differences increased parabolically with lesion size on the linear scale (Figure 2). To average out the readers, we calculated averages from each pair of measurements and computed the ratio of “size after treatment” over “size before treatment” for each lesion. The resulting stalactite plots visualize the response of each patient sorted by the mean response of all lesions (Figure 3). Notably, lesion size response did not correlate with the initial lesion size at baseline imaging (Pearson’s r = 0.144; 95% confidence interval 0–0.324; Supplemental Figure S1). The histogram of all lesion responses, having a peak near 100% (no response) and a pronounced tail to the left, suggests the presence of both clearly responding and essentially stable lesions (Supplemental Figure S2). Table 2 details lesion size per patient before and after E-KRd induction therapy. On a patient level, the mean overall lesion size reduction was calculated to be 22% (95% confidence interval: 14–31%; *p* < 0.001). Figure 4 includes an exemplary case of substantial lesion size decrease and trabecular sclerosis.

### 3.3. Qualitative Criteria of Therapy Response

**Figure 5:**
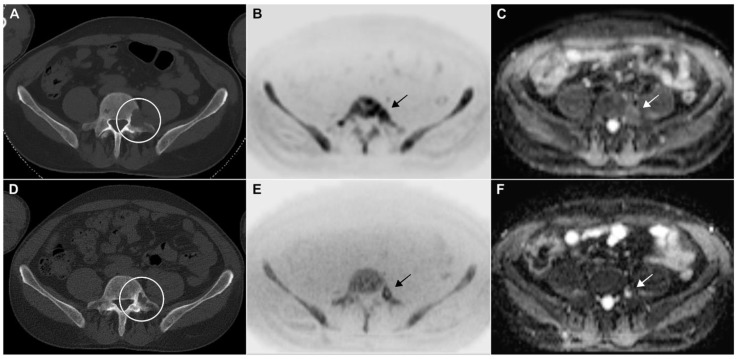
Baseline CT in a 48-year-old man with newly diagnosed multiple myeloma (IgG lambda, R-ISS I) depicted a large osteolysis in lumbar vertebra 5 ((**A**): circle) with active tumor signal in diffusion-weighted MRI ((**B**): inverted gray scale image with b-value of 2000 s/mm^2^; (**C**): ADC map; arrows). After six cycles of E-KRd, the lesion was significantly smaller, exhibiting considerable remineralization ((**D**): circle). Hypocellular matrix and high ADC signal in MRI ((**E**,**F**): arrows).

**Figure 6:**
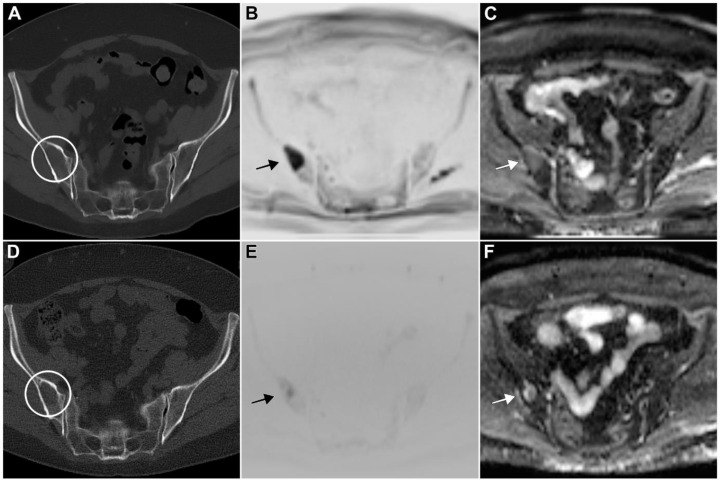
58-year-old woman with multiple myeloma (IgG kappa, R-ISS I) before (upper row) and after six cycles of E-KRd (lower row). Axial low-dose CT showed an osteolysis in the right iliac crest without size decrease or sclerosis after treatment ((**A**,**D**): circles). Signs of therapy response in diffusion-weighted MRI with residual disease ((**B**,**E**): low signal in inverted gray scale images with b-value of 2000 s/mm^2^; (**C**,**F**): incomplete ADC signal increase; arrows).

Inter-reader reliability was high for the presence of cortical destruction (α = 0.85; 95% confidence interval 0.76–0.93), pathologic fractures (0.92; 0.81–1), rim sclerosis (0.74; 0.64–0.83) and trabecular remineralization (0.81; 0.68–0.91). Therefore, we chose to aggregate the binary features over readers, looking only at lesions concordantly called by both radiologists at both time points (*n* = 94; for cross-tabulations see Supplemental Tables S2 and S3). At baseline imaging, cortical destruction was ascertained by the two radiologists in 33 osteolytic manifestations (35%), while pathologic fractures were determined in five lesions (5%). Cortical destruction at the time of follow-up was described in 12 lesions (13%), while three new pathologic fractures emerged. On baseline scans, rim sclerosis was found in nine lesions (10%), whereas no trabecular sclerosis was detected. After treatment, the number of MM manifestations exhibiting rim sclerosis increased to 51 (54%), while trabecular remineralization was ascertained by both readers in 29 lesions (31%). The reader-specific qualitative lesion characterization is summarized in Table 3. Logistic regression analysis revealed that trabecular sclerosis in at least one lesion was strongly associated with a substantial overall patient-level response (Supplemental Figure S3; *p* ≤ 0.001). Accordingly, among lesions showing a size reduction of more than 20%, trabecular sclerosis was recorded in 53% (Table 4). Extensive bone healing in a previously vital MM manifestation with cortical destruction at baseline is depicted in Figure 5, whereas Figure 6 shows a patient without bone remineralization despite signs of therapy response after induction therapy.

## 4. Discussion

This study retrospectively investigated bone remineralization in patients with newly diagnosed multiple myeloma under a quadruple induction therapy of carfilzomib, lenalidomide, dexamethasone and the anti-SLAMF7 monoclonal antibody elotuzumab. We validated the measurement of osteolytic lesion size as a reliable bone remineralization study endpoint and showed that the presence of trabecular sclerosis is strongly associated with a positive response, suggesting its potential as a radiological marker for treatment efficacy and bone remineralization in MM patients. The absence of correlation between lesion size response and initial lesion size underscores the potential of using E-KRd in treating lesions of varying sizes, potentially improving patient outcomes across the spectrum of disease severity.

Represented in the IMWG SliM-CRAB criteria for MM defining events, the primary function of radiology in patients with MM lies in the detection of bone marrow manifestations (M = MRI with more than one focal lesion larger than 5 mm) and osteolyses (B = bone disease; one or more osteolytic lesion). For the latter, whole-body low-dose CT is the most established means of diagnosis due to fast image acquisition, ubiquitous availability and excellent diagnostic sensitivity [4]. Although the main strength of CT imaging lies in the detection of osteolytic lesions [5,7], the process of remineralization under different therapy regimens has only been investigated sporadically for disease monitoring thus far [22,23,24].

In the last two decades, the introduction of new therapeutic agents, such as proteasome inhibitors and immunomodulators, has altered the outcome and overall survival rates of MM patients in different stages of disease [25,26,27]. Nonetheless, diffuse and focal bone demineralization continues to negatively affect individuals’ quality of life. Particularly, the increased risk for pathologic fractures remains a major driver of morbidity and mortality in MM patients [9]. In a previous study on bone remineralization in MM, Schulze et al. described sclerosis in only 18% of patients receiving bortezomib treatment; however, lesion size was not investigated by the authors, and no difference was identified between rim and trabecular sclerosis [14]. While the rate of remineralization was higher in the present study, our results also indicate a high degree of agreement between readers, which is decisive for clinical routine, since different radiologists will most likely be involved in the CT examinations of a single patient over years of treatment [14]. With 43% sclerosis, the proportion of remineralization in lytic lesions of the pelvis region reported by Mohan et al. was more in line with our findings [22], despite manifestations being significantly larger than in the present study (average of 4 cm). In a more recent analysis, the same authors even determined a 72% rate of remineralization over various anatomical regions, albeit only evaluating one focal osseous lesion per patient [28]. Of note, all study samples reported in the literature consist of patients in various stages of disease instead of a homogenous population of patients with newly diagnosed MM. Further limiting comparisons with our results, patients in earlier studies received chemotherapy according to the treatment protocols of various different clinical trials instead of a singular therapy regimen such as E-KRd.

The following limitations must be considered for this study. First, the study did not include a control group receiving an established induction regimen, since the current investigation was performed to assess the general process of bone remineralization in a homogenous group of newly diagnosed MM patients instead of specifically analyzing the therapy effect of E-KRd. However, upcoming prospective studies should use the validated study endpoint to compare the effects of different immunomodulatory agents and also evaluate their combination with exercise therapy concepts. Of note, the latter have already shown encouraging effects in MM and monoclonal gammopathy patients and may be a focal point of future research on bone health [29,30]. Second, the investigated population consisted solely of patients with newly diagnosed MM. While patients with relapsed/refractory MM may display a different extent of bone remineralization under treatment, studies investigating the effect of the human monoclonal antibody daratumumab in such samples have also revealed improved bone turnover [31,32]. Third, while the proportion of lesions developing rim or trabecular sclerosis as a form of therapy response was high in our cohort, the overall number of patients eligible for study inclusion was rather small, since we prioritized homogeneity within the patient sample over a less standardized population. In concordance with the study’s aim, we believe that the standardization of therapy and diagnostics is essential to establish a valid and reliable endpoint for any form of treatment response.

## 5. Conclusions

The sum of osteolytic lesion sizes on sequential whole-body low-dose CT scans defines a reliable patient-level study endpoint to characterize bone remineralization. Patient-level response was strongly associated with the presence of trabecular sclerosis. As the sum of lesion sizes defines a valid study endpoint to characterize remineralization on a patient level, this endpoint can be used to quantify bone health during different forms of therapy.

## Figures and Tables

**Table 1:** Study population.

Patient	Age	Sex	Diagnosis	R-ISS	E-KRd Cycles	Bone Therapy (q4w)	Interval between CT Scans (days)	Bone Marrow Infiltration at Baseline (%)	Bone Marrow Infiltration after Treatment (%)	Hematologic Response at End of E-KRd
1	52	m	IgG lambda	II	6	Denosumab	209	90	<10	VGPR
2	61	m	IgG kappa	I	6	Zoledronic acid	204	90	3	VGPR
3	54	m	IgG lambda	II	6	Zoledronic acid	245	20	0	VGPR
4	69	f	IgG kappa	I	6	Zoledronic acid	207	50	0	VGPR
5	58	m	IgG kappa	II	6	Zoledronic acid	218	70	<10	VGPR
6	59	f	IgG kappa	I	6	Zoledronic acid	214	30	0	VGPR
7	48	m	IgG lambda	I	6	Zoledronic acid	294	0	0	VGPR
8	58	f	IgG kappa	I	6	Zoledronic acid	202	70	0	VGPR
9	57	m	IgG kappa	I	6	Zoledronic acid	198	8	0	CR
10	58	m	IgG kappa	II	6	Zoledronic acid	230	70	0	VGPR
11	62	f	IgG kappa	I	6	Denosumab *	204	15	0	VGPR
12	56	m	IgA kappa	II	6	Zoledronic acid	205	90	0	VGPR
13	57	m	IgG kappa	II	6	Zoledronic acid	280	90	0	VGPR
14	63	m	IgG lambda	I	6	Zoledronic acid	209	20	<10	VGPR
15	40	m	IgA kappa	I	6	Zoledronic acid	229	30	0	CR
16	58	f	IgA kappa	I	6	Zoledronic acid	211	80	10	VGPR
17	69	m	IgA lambda	I	6	Pamidronic **	207	15	0	CR
18	41	f	IgG kappa	II	6	Zoledronic acid	242	45	0	sCR
19	68	m	IgG kappa	III	6	Zoledronic acid	217	60	<10	VGPR
20	66	f	IgG lambda	II	6	Zoledronic acid	196	50	5	VGPR

**Note.** Zoledronic acid 4 mg (intravenous) with drug dose adjustment for renal function at baseline or denosumab 120 mg (subcutaneous) were applied every 4 weeks. * denosumab 60 mg every 6 months (subcutaneous); ** pamidronic acid every 3 months (intravenous). CR—complete response; E-KRd—treatment with elotuzumab, carfilzomib, lenalidomide, and dexamethasone; R-ISS—Revised International Staging System; sCR—stringent complete response; VGPR—very good partial response.

**Table 2:** Therapy response based on reader-aggregated lesion size per patient.

Patient	Number of Lesions	Sum of Lesion Sizes at Baseline (mm^2^)	Sum of Lesion Sizes after Treatment (mm^2^)	Sum of Response Ratios	Mean Response (%)
1	6	1108	818	0.74	−25
2	4	912	811	0.89	−3
3	9	1877	1506	0.80	−20
4	5	933	526	0.56	−37
5	8	2238	1706	0.76	−25
6	5	542	530	0.98	+2
7	10	1136	498	0.44	−58
8	5	510	496	0.97	−4
9	4	621	249	0.4	−82
10	4	492	402	0.82	−38
11	9	1320	910	0.69	−51
12	3	808	692	0.86	−15
13	6	2040	1726	0.85	−15
14	2	104	56	0.54	−35
15	6	640	632	0.99	−1
16	4	477	433	0.91	−24
17	5	745	710	0.95	−7
18	2	212	198	0.93	−8
19	5	2291	1659	0.72	−35
20	6	766	554	0.72	−36

**Note.** Measurement aggregation performed per lesion in each patient over the two readers on the square root scale.

**Table 3:** Qualitative osteolytic lesion characterization.

Reader Analysis	Lesions Described by Reader 1 (*n* = 103)		Lesions Described by Reader 2 (*n* = 99)		Lesions Described by Reader 1 and 2 (*n* = 94)		Inter-Reader Reliability
CT Scan	Before Treatment	After Treatment	Before Treatment	After Treatment	Before Treatment	After Treatment	Krippendorff’s α
Cortical destruction	35% (36/103)	17% (17/103)	37% (37/99)	12% (12/99)	35% (33/94)	13% (12/94)	0.85 (0.76–0.93)
Pathologic fracture	6% (6/103)	9% (9/103)	5% (5/99)	8% (8/99)	5% (5/94)	9% (8/94)	0.92 (0.81–1)
Rim sclerosis	14% (14/103)	72% (74/103)	9% (9/99)	56% (55/99)	10% (9/94)	54% (51/94)	0.74 (0.64–0.83)
Trabecular sclerosis	0% (0/103)	33% (34/103)	1% (1/99)	39% (39/99)	0% (0/94)	31% (29/94)	0.81 (0.68–0.91)

**Note.** For binary features, the numbers given first indicate relative frequencies (%), while absolute numbers of items are displayed in parentheses. As a measure of inter-reader agreement, Krippendorff’s α is presented with 95% confidence intervals in parentheses.

**Table 4:** Therapy response and trabecular sclerosis. Among lesions displaying a response greater than 20% after E-KRd induction therapy, trabecular sclerosis was ascertained in 53% (24/45). In contrast, only 8% (5/63) of lesions with a treatment response equal to or less than 20% depicted suchlike forms of remineralization.

Lesion Level Response	No Trabecular Sclerosis	Trabecular Sclerosis	Sum	*p* Value
Therapy response ≤20%	58 (54%)	5 (5%)	63 (58%)	
Therapy response >20%	21 (19%)	24 (22%)	45 (42%)	
Sum	79 (73%)	29 (27%)	108 (100%)	<0.001 *

**Note.** All 108 lesions described after E-KRd induction therapy were aggregated in this contingency table to calculate the response and trabecular sclerosis ratios. Fisher’s exact test was performed for the assessment of statistical significance (*).

## Data Availability

The datasets generated and/or analyzed during this study are not publicly available as CT data and DICOM headers contain patient information. Data can be obtained on reasonable request from the corresponding author.

## Supplementary Materials

The following supporting information can be downloaded at: https://www.mdpi.com/article/10.3390/cancers15154008/s1; Figure S1: Correlation of lesion response with baseline size; Figure S2: Mean response histogram; Figure S3: Logistic regression analysis; Table S1: E-KRd induction therapy protocol; Table S2: Reader concordance on bone destruction; Table S3: Reader concordance on bone remineralization.

## Abbreviations

E-KRd	elotuzumab, carfilzomib, lenalidomide, and dexamethasone
IMWG	International Myeloma Working Group
MM	multiple myeloma
(s)CR	(stringent) complete response
SLAMF-7	signaling lymphocytic activation molecule family member 7
VGPR	very good partial response
