# Supplementary material for: Assessing Osteolytic Lesion Size on Sequential CT Scans Is a Reliable Study Endpoint for Bone Remineralization in Newly Diagnosed Multiple Myeloma
Source: Cancers (Basel). 2023 Aug 7;15(15):4008. doi: 10.3390/cancers15154008 (PMC10417114; doi:10.3390/cancers15154008)
Supplement: Supplementary file 1 [file cancers-15-04008-s001.zip › Supplemental Figures S1-S3.pdf]

Supplemental Figure

Supplemental Figure Legends

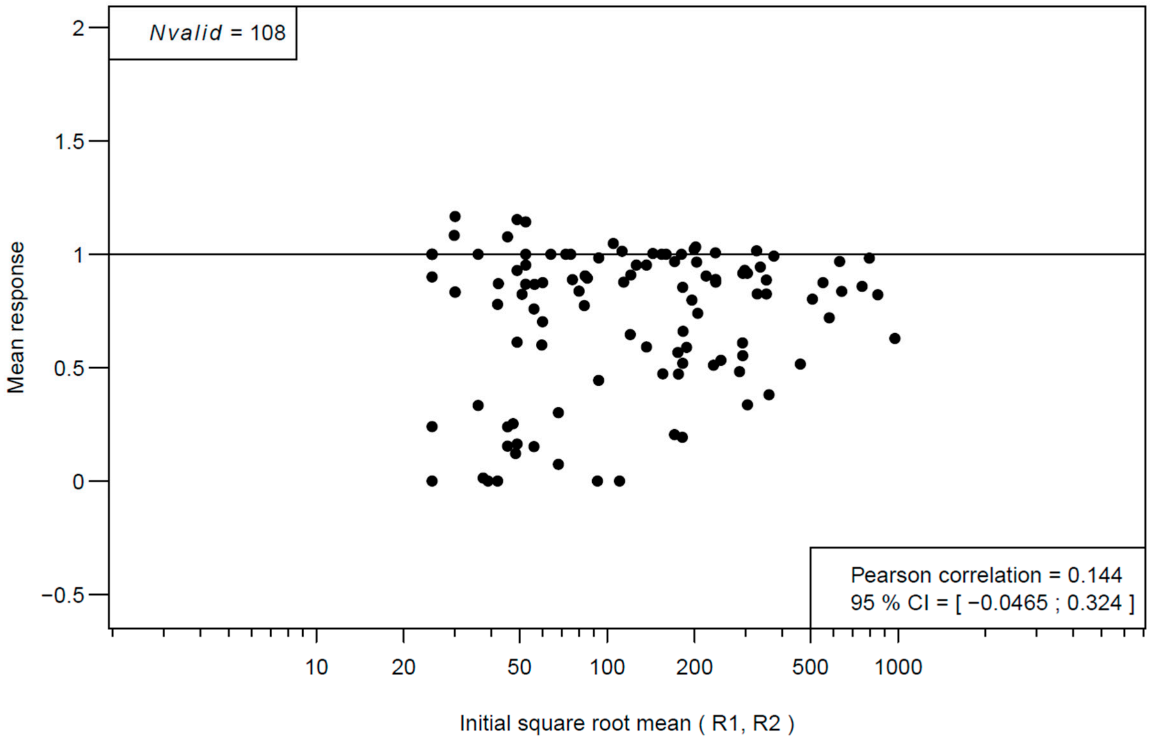

Supplemental Figure S1

The graph illustrates that therapy response after E-KRd induction does not correlate substantially with lesion size at baseline imaging.

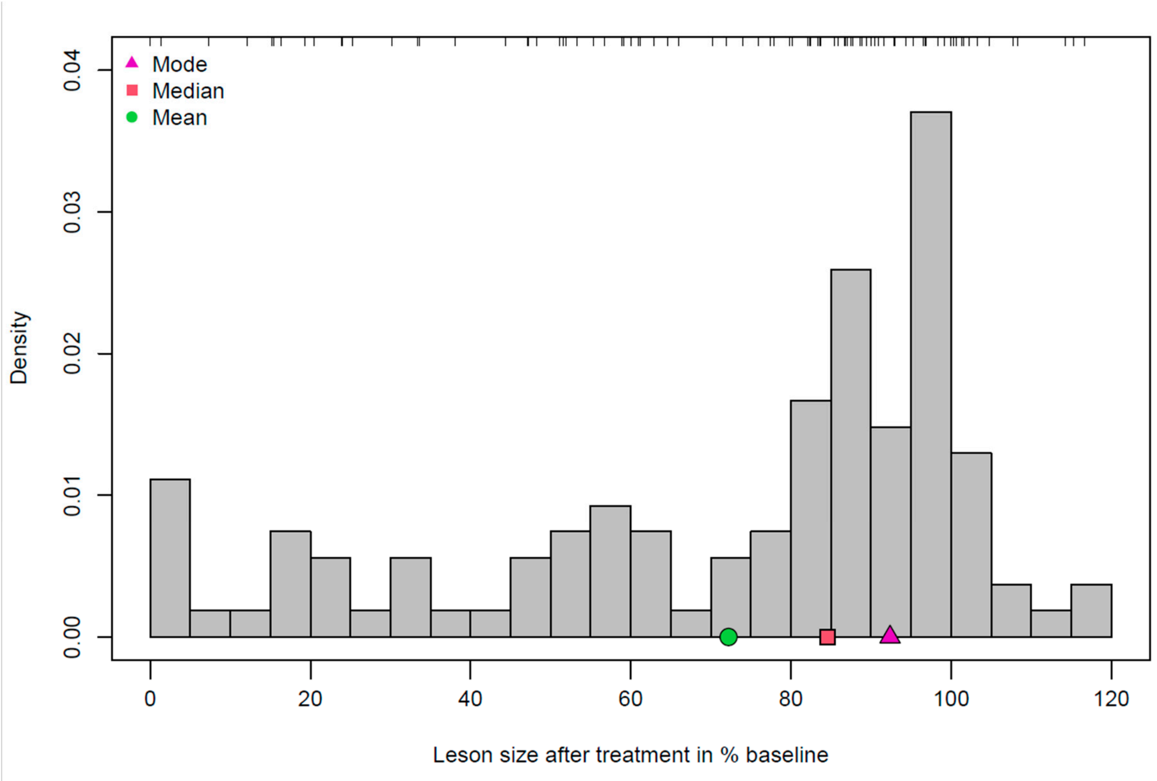

<u>Supplemental Figure S2</u>

The mean response histogram has a peak near 100% on the *x*-axis and a pronounced tail to the left, suggesting the presence of clearly responding lesions (>20% reduction) and essentially stable osteolyses.

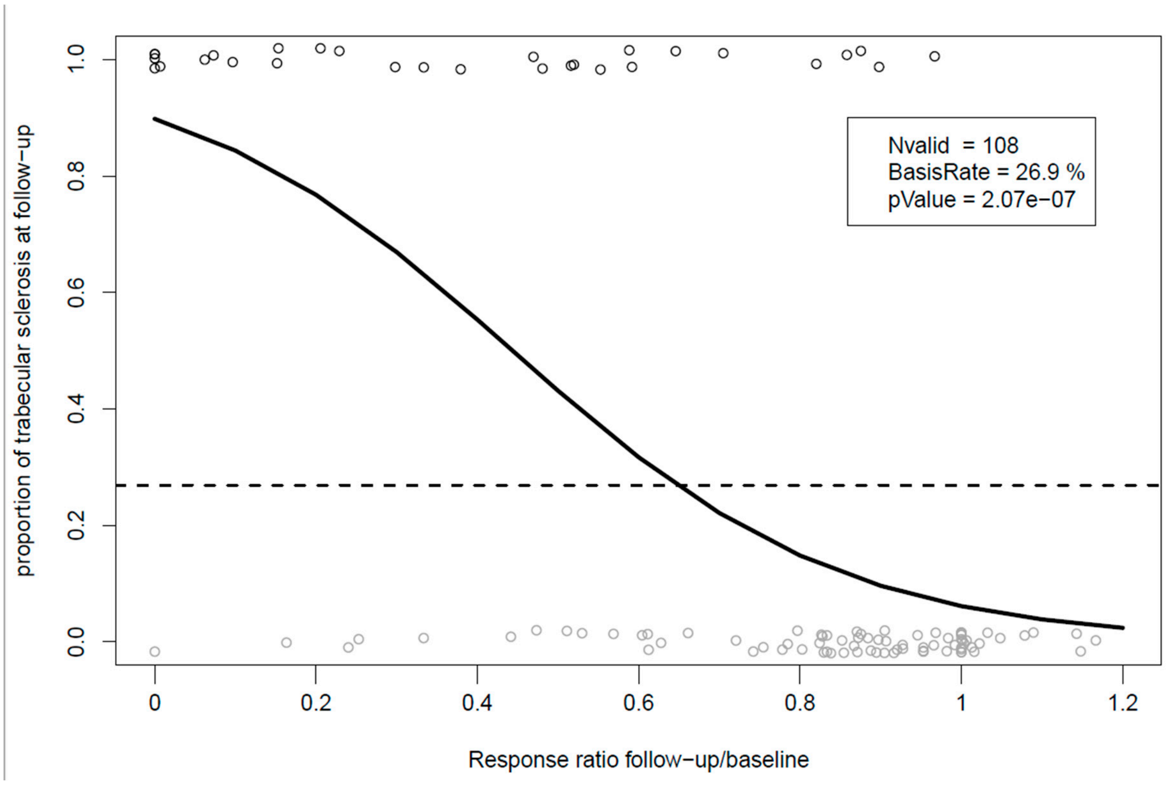

<u>Supplemental Figure S3</u>

In logistic regression analysis, trabecular sclerosis proved to be strongly associated with lesion response in size ($p<0.001$).
