# Supplementary material for: Assessing Osteolytic Lesion Size on Sequential CT Scans Is a Reliable Study Endpoint for Bone Remineralization in Newly Diagnosed Multiple Myeloma
Source: Cancers (Basel). 2023 Aug 7;15(15):4008. doi: 10.3390/cancers15154008 (PMC10417114; doi:10.3390/cancers15154008)
Supplement: Supplementary file 1 [file cancers-15-04008-s001.zip › Supplemental Table S1.pdf]

Supplemental Table S1 – E-KRd induction therapy protocol

Therapy	Dosage	Induction
**Elotuzumab** (intravenous infusion)	10 mg/kg	Day 1, 8, 15, 22 of cycle 1 and 2,
	10 mg/kg	Day 1 and 15 of cycles 3-6.
**Carfilzomib** (intravenous infusion)	20 mg/m²	Day 1 and 2 of cycle 1
	36 mg/m²	Day 8, 9, 15, 16 of cycle 1
	36 mg/m²	Day 1, 2, 8, 9, 15, 16 of cycle 2-6
**Lenalidomide** (oral hard capsule)	25 mg	Day 1-21 of cycle 1-6
**Dexamethasone** (oral and intravenous)	28 mg oral and 8 mg intravenous	Day 1, 8, 15, 22 of cycles 1-2
	28 mg oral and 8 mg intravenous	Day 1 and 15 of cycles 3-6
	40 mg oral	Day 8 and 22 of cycle 3-6
