# Supplementary material for: Assessing Osteolytic Lesion Size on Sequential CT Scans Is a Reliable Study Endpoint for Bone Remineralization in Newly Diagnosed Multiple Myeloma
Source: Cancers (Basel). 2023 Aug 7;15(15):4008. doi: 10.3390/cancers15154008 (PMC10417114; doi:10.3390/cancers15154008)
Supplement: Supplementary file 1 [file cancers-15-04008-s001.zip › Supplemental Table S2.pdf]

Supplemental Table S2 – Reader concordance on bone destruction

Concordance for the presence of cortical destruction in lesions suspected by at least one of the readers was 80% (45/[45+7+4]). For pathologic fractures, reader concordance was 87% (13/[13+2+0]).

**Cortical destruction**		**Reader 2**		
		Absent	Present	Sum
	Absent	132 (70%)	4 (2%)	136 (72%)
**Reader 1**	Present	7 (4%)	45 (24%)	52 (28%)
	Sum	139 (74%)	49 (26%)	188 (100%)
**Pathologic fracture**		**Reader 2**		
		Absent	Present	Sum
	Absent	173 (92%)	0 (0%)	173 (92%)
**Reader 1**	Present	2 (1%)	13 (7%)	15 (8%)
	Sum	175 (93%)	13 (7%)	188 (100%)

Note. *– Concordance was calculated for all 188 lesions described by both readers at baseline and follow-up imaging.*
