# Supplementary material for: Assessing Osteolytic Lesion Size on Sequential CT Scans Is a Reliable Study Endpoint for Bone Remineralization in Newly Diagnosed Multiple Myeloma
Source: Cancers (Basel). 2023 Aug 7;15(15):4008. doi: 10.3390/cancers15154008 (PMC10417114; doi:10.3390/cancers15154008)
Supplement: Supplementary file 1 [file cancers-15-04008-s001.zip › Supplemental Table S3.pdf]

Supplemental Table S3 – Reader concordance on bone remineralization

Concordance for the presence of rim sclerosis in lesions suspected by at least one of the readers was 72% (60/[60+21+2]). For trabecular sclerosis, reader concordance was 73% (29/[29+3+8]).

**Rim sclerosis**		**Reader 2**		
		Absent	Present	Sum
**Reader 1**	Absent	105 (56%)	2 (1%)	107 (57%)
	Present	21 (11%)	60 (32%)	81 (43%)
	Sum	126 (67%)	62 (33%)	188 (100%)

**Trabecular sclerosis**		**Reader 2**		
		Absent	Present	Sum
**Reader 1**	Absent	148 (79%)	8 (4%)	156 (83%)
	Present	3 (2%)	29 (15%)	32 (17%)
	Sum	151 (80%)	37 (20%)	188 (100%)

Note. *– Concordance was calculated for all 188 lesions described by both readers at baseline and follow-up imaging.*
